# Impact of Pulmonary Arterial Hypertension on Employment, Work Productivity, and Quality of Life - Results of a Cross-Sectional Multi-Center Study

**DOI:** 10.3389/fpsyt.2021.781532

**Published:** 2022-02-08

**Authors:** Jan Fuge, Da-Hee Park, Thomas von Lengerke, Manuel J. Richter, Henning Gall, Hossein A. Ghofrani, Jan C. Kamp, Marius M. Hoeper, Karen M. Olsson

**Affiliations:** ^1^Department of Respiratory Medicine, Hannover Medical School, Hannover, Germany; ^2^Biomedical Research in Endstage and Obstructive Lung Disease Hannover, German Center for Lung Research, Hannover, Germany; ^3^Department of Medical Psychology, Centre for Public Health and Healthcare, Hannover Medical School, Hannover, Germany; ^4^Department of Internal Medicine, Justus Liebig University Giessen, Universities of Giessen and Marburg Lung Center, Giessen, Germany; ^5^The German Center for Lung Research, Giessen, Germany

**Keywords:** employment status, work productivity, work productivity activity impairment questionnaire, pulmonary arterial hypertension, quality of life, social participation, education

## Abstract

**Introduction:**

Data on burden of pulmonary arterial hypertension (PAH) are mostly limited to physical and clinical endpoints as well as quality of life. Research on employment, work productivity, and educational background is scarce. The aim of this study was to assess the impact of PAH on employment status and work productivity in Germany.

**Materials and Methods:**

In a multicenter cross-sectional survey, patients with PAH were surveyed in two large pulmonary hypertension referral centers in Germany. The survey contained questions on education, employment, work productivity and impairment (WPAI, also at the time of diagnosis), quality of life, and socioeconomic status. Additional data was assessed using clinical research database for 6-min walk distance (6MWD), WHO functional class, and N-terminal fragment of pro-brain natriuretic peptide. All patients provided written informed consent, and the institutional review board approved this study.

**Results:**

In total, 212 patients were surveyed (72% female; median, 57 years) approximately 6 years after initial PAH diagnosis. A total of 76% had an idiopathic PAH followed by hereditary and associated PAH (10% each). Employment at the time of diagnosis was 48% (34% full-time, 14% part-time), with productivity measured by a WPAI score of 6 points and decreased to 29% (21% full-time, 8% part-time) at the time of the survey (with a WPAI score of 2 points, *p* < 0.001). Logistic regression showed education and 6MWD as predictors for employment. Patients in moderate or high educational category had a 3.6- or 5.6-fold chance, respectively, of being employed (*p* = 0.025 and *p* = 0.019), and per 50-m increase of 6MWD, the odds of being employed were 1.2, *p* = 0.042.

**Conclusion:**

Patients with PAH had a reduced employment rate, which was influenced by education and 6MWD. There was a considerable loss of employment over the course of the disease. Employment should be one possible treatment goal in patients with PAH to provide social participation to this patient group.

## Introduction

Pulmonary arterial hypertension (PAH) is a rare pulmonary disease marked by debilitating symptoms, such as exertional dyspnea, syncope, and clinical signs of heart failure (1). PAH is characterized by pulmonary vascular remodeling, leading to increased pulmonary vascular resistance. Despite successful therapeutic strategies, PAH is frequently fatal, with right heart failure being the leading cause of death (2, 3). Patients with PAH experience impaired physical activity and quality of life (QoL) (4). Sociodemographic data on PAH patients is scarce. To our knowledge, no study addressed the factors influencing the status of employment in patients with PAH so far.

The impact on work productivity of numerous diseases, including asthma, irritable bowel syndrome, and rheumatoid arthritis, has been widely studied (5, 6). Research included missed time from work, reduced performance, and reduced routine working hours. Reilly et al. developed the work productivity and impairment (WPAI) questionnaire to assess the effect of health and symptom severity on work productivity (7).

While the burden of PAH with its limitations on physical capability and QoL is widely recognized, employment, work productivity, and educational background remain underrepresented (8). There are few studies exclusively discussing the employment status of patients with PAH. Taichman et al. suggest that better physical QoL is associated with active employment and found an employment rate of 35% (1). Another study demonstrated the symptoms of PAH to impair the ability to perform at work and maintain a full-time work schedule and lead to feelings of loss of independence and purpose (9). In the US, Matura et al. found a diminished rate of employment in PAH patients and the educational status to be a strong predictor for overall QoL (10). The European Pulmonary Hypertension Association surveyed 326 patients and found that employment and work are main issues for at least 85% of patients with PAH, resulting in a relevant loss of income (11). So far, no data exist on work productivity or employment status for patients with PAH in Germany. However, there is some data on employment and QoL in patients with left heart failure. With high rates of retired patients, just 9 to 15% of the patients were employed. The employed patients reported 29% impaired work productivity (12, 13).

The aim of this study was to assess the impact of PAH on employment over the course of the disease as part of social participation. Factors associated with not being able to work were analyzed. The secondary outcomes were QoL in relation to employment, work productivity, and clinical status of PAH patients.

## Materials and Methods

In this cross-sectional, observational, multicenter study, patients with a confirmed diagnosis of PAH at two participating pulmonary hypertension (PH) referral centers (Hannover Medical School and University of Giessen, both in Germany) were surveyed. This study was conducted between November 2018 and October 2019 in routine outpatient care using a paper-based self-administered questionnaire. The study was approved by the local institutional review board (no. 8115_BO-K_2018), and all patients gave written informed consent for the use of their data.

### Patient Setting and Clinical Parameters

All patients in both centers with a confirmed diagnosis of PAH according to current European Society of Cardiology/European Respiratory Society (ESC/ERS) Guidelines (2) and with age ≥18 years were invited to complete a questionnaire as part of their routine outpatient visit. For clinical characterization, hemodynamics from right heart catheterization at time of diagnosis were assessed. The study visit included WHO functional class (FC), 6-min walk distance (6MWD), serum levels of N-terminal fragment of pro-brain natriuretic peptide (NT-proBNP), anthropometry (body weight, height, and body mass index), and pulmonary function testing (PFT). PFT included forced expiratory volume in 1 s (FEV1) and diffusing capacity of the lung for carbon monoxide (D_LCO_). The ERS/ESC risk score assessment for our cohort was based on three variables (FC, 6MWD, and BNP/NT-proBNP) as previously described (14, 15). Each variable was graded with a number from 1 to 3, representing low risk = 1, intermediate risk = 2, and high risk = 3. The average risk was calculated by dividing the sum of the grades by the number of available variables and rounding to the next integer.

### Assessment of Socioeconomic Status, Lifestyle Factors, Work Productivity, and HRQoL

We assessed socioeconomic status (employment and education) as well as work productivity and health-related quality of life. Employment status and work productivity were assessed both for the time of diagnosis (retrospectively) and at study visit (current status). The sociodemographic standards of the German Federal Statistical Office (Destatis) (16) were used to assess employment as well as general and professional education. Educational status was further categorized in low, moderate, and high education as follows: no education (low), non-academic education (moderate), and academic education (high). Education was compared to the German population using the German population census “Mikrozensus 2018,” and retirement types (due to age or due to disability) were compared using data from the German retirement agency (Deutsche Rentenversicherung Bund) (17, 18). Work productivity was assessed using the WPAI instrument (7). Focusing on question #5 of WPAI, the patients were asked by how much their productivity, while working during the past 7 days, was affected by PAH-derived health problems. The patients were asked to encircle a number between zero and 10, ranging from health problems had no effect on work productivity to health problems completely prevented the patient from working. The patients were asked to omit this question in case they were not employed.

At study visit, QoL, family status, degree of disability, smoking status, and alcohol use were assessed. QoL was assessed using the EuroQoL EQ-5D sub-scored in EQ-5D-3L and EQ-VAS (19) and emPHasis-10 (20). Details for the instruments used are found in Supplementary Table 1. The EQ-5D-3L has five dimensions (mobility, self-care, usual activities, pain/discomfort, and anxiety/depression). Higher scores indicate a better quality of life. The EQ-VAS is a scale with a range from 0 (the worst health you can imagine) to 10 (the best health you can imagine). The emPHasis-10 is a PH-specific QoL questionnaire. The emPHasis-10 score ranges from 0 to 50, with higher scores indicating a worse QoL. The degree of disablement in Germany is a score between 20 and 100 points, characterizing the extent of disability of a patient, with a higher score indicating a greater disability. The patients were asked to state their smoking status (active smoker, former smoker, or never a smoker) as well as the duration of smoking and the mean number of cigarettes per day to calculate the number of packyears (21).

### Statistical Analysis

IBM SPSS Statistics (version 27.0, IBM Corp., Armonk, NY, USA) and Stata 13.0 (State Corp LP, College Station, TX, USA) statistical software programs were used for statistical analysis. Categorical data were presented as counts with percentages. Continuous variables were presented as median with the first and third quartile (Q1 and Q3) or as mean and standard deviation (SD), depending on the distribution. For group comparisons, chi-square test, Fisher's exact test, or McNemar–Bowker test, in case of paired comparison, was used as appropriate. Continuous parameters were compared using paired *t*-test, or Wilcoxon test was used as appropriate. To assess the impact on employment in patients who were eligible of being employed (using the official German retirement age of 67 years as the cutoff), simple and multiple logistic regression analysis was conducted (forward-stepwise conditional) and odds ratios (OR) were calculated. Significant variables of the simple model were included in the multiple model, and variables with *p* < 0.10 remained in the multiple model. To visualize change in categories over time, Sankey–Chart diagrams were created. All tests were two-sided, with *p* < 0.05 considered to be statistically significant.

## Results

**Figure 1:**
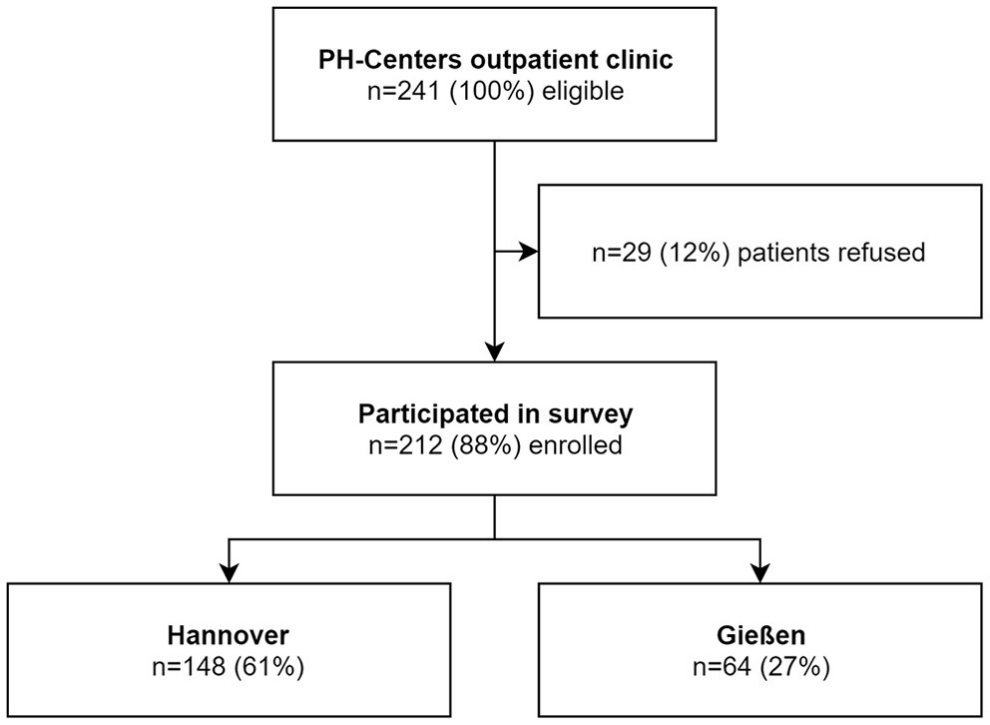
Flow chart of study inclusion. PH, pulmonary hypertension.

A total of 212 from 241 (88%) patients agreed to participate and returned their questionnaires; 148 (70%) were from Hannover and 64 (30%) were from Gießen (see Figure 1). Female sex comprised 72%, and the majority of the patients (76%) had a group 1.1 idiopathic PAH, followed by hereditary PAH, and associated PAH (10% each; see Table 1). The median age at study visit was 57 (43–66) years, and the study visit occurred 6 (2–11) years after the diagnosis of PAH.

**Table 1:** Patient characteristics at study visit.

	**Demographics, *n* = 212 patients**
**Age in years, median (IQR)**	57 (43–66)
Sex, *n* (%)	
Female	2%)
Male	59 (28%)
Diagnosis, *n* (%)	
IPAH—group 1.1	(6%)
HPAH—group 1.2	(10%)
DPAH—group 1.3	(2%)
APAH—group 1.4	(10%)
PVOD/PCH—group 1.6	3 (1%)
**Age at diagnosis in years, median (IQR)**	48 (34–60)
**Time since diagnosis in years, median (IQR)**	6 (2–11)
Hemodynamic at diagnosis, median (IQR)	
mPAP (mmHg)	50 (42–57)
PVR (dyn s cm^−5^)	767 (543–1,031)
Cardiac index (L/min/m^2^)	2.3 (1.9–2.7)
WHO FC, *n* (%)	
I	22 (10%)
II	(37%)
III	(8%)
IV	10 (5%)
PAH medication, *n* (%)	
Monotherapy	(28%)
Double combination therapy	(45%)
Triple combination therapy	55 (26%)
**NT-proBNP (ng/L), median (IQR)**	221 (92–611)
ESC/ERS risk score	*n* = 169, 43 missing values (20%)
Low	(55%)
Intermediate	(41%)
High	6 (4%)
Pulmonary function test, median (IQR)	
FEV_1_ (% of predicted)	(65–90)
D_LCO_ (% of predicted)	60 (49–71)
6MWD, median (IQR)	
Walk distance (m)	462 (369–546)
Flights of stairs, *n* (%)	*n* = 204, 8 missing values (4%)
None	25 (12%)
One flight	89 (44%)
Two flights or more	90 (44%)
**BMI (kg/m** ^**2**^ **), median (IQR)**	26 (23–31)
Smoking status, *n* (%)	*n* = 207, 5 missing values (2%)
Active smoker	30 (15%)
Former smoker	73 (35%)
Never a smoker	104 (50%)
**Packyears, median (IQR)**	13 (6–26)
Education, *n* (%)	*n* = 206, 6 missing values (3%)
Low education	35 (17%)
Moderate education	134 (63%)
Higher education	37 (18%)
HRQoL scores	
EQ-5D-3L, median (IQR)	7 (6–9)
EQ-VAS, median (IQR)	5.5 (3.5–7.5)
emPHasis-10, median (IQR)	18 (13–27)

*IQR, interquartile range; PH, pulmonary hypertension; IPAH, idiopathic pulmonary hypertension; HPAH, hereditary pulmonary hypertension; APAH, associated pulmonary hypertension; DPAH, drug- and toxin-induced pulmonary hypertension; PVOD, pulmonary veno-occlusive disease; PCH, pulmonary capillary hemangiomatosis; BMI, body mass index; MPAP, mean pulmonary arterial pressure; PVR, pulmonary vascular resistance; WHO-FC, World Health Organization functional class; NT-proBNP, N-terminal fragment of probrain natriuretic peptide; ESC, European Society of Cardiology; ERS, European Respiratory Society; FEV1, forced expiratory volume in 1 s; DLCO, diffusing capacity of the lung for carbon monoxide; 6MWD, 6-min walk distance; AUDIT-C, Alcohol Use Disorder Identification Test; VAS, Visual Analogue Scale*.

Most patients were in WHO FC II or III (85%) and in low-risk ESC/ERS category (55%), and 44% were capable to climb a maximum of one flight of stairs. PFT showed a slight reduction in FEV_1_ (78% predicted) and a moderate reduction in D_LCO_ (60% predicted). The NT-proBNP was 221 ng/L (92–611), and 6MWD was 448 m (338–540). Fifty percent of the patients had a history of smoking, with a median of 13 (6–26) packyears, of whom 15% were active smokers.

### Education, Employment, Work Productivity, and HRQoL

**Figure 2:**
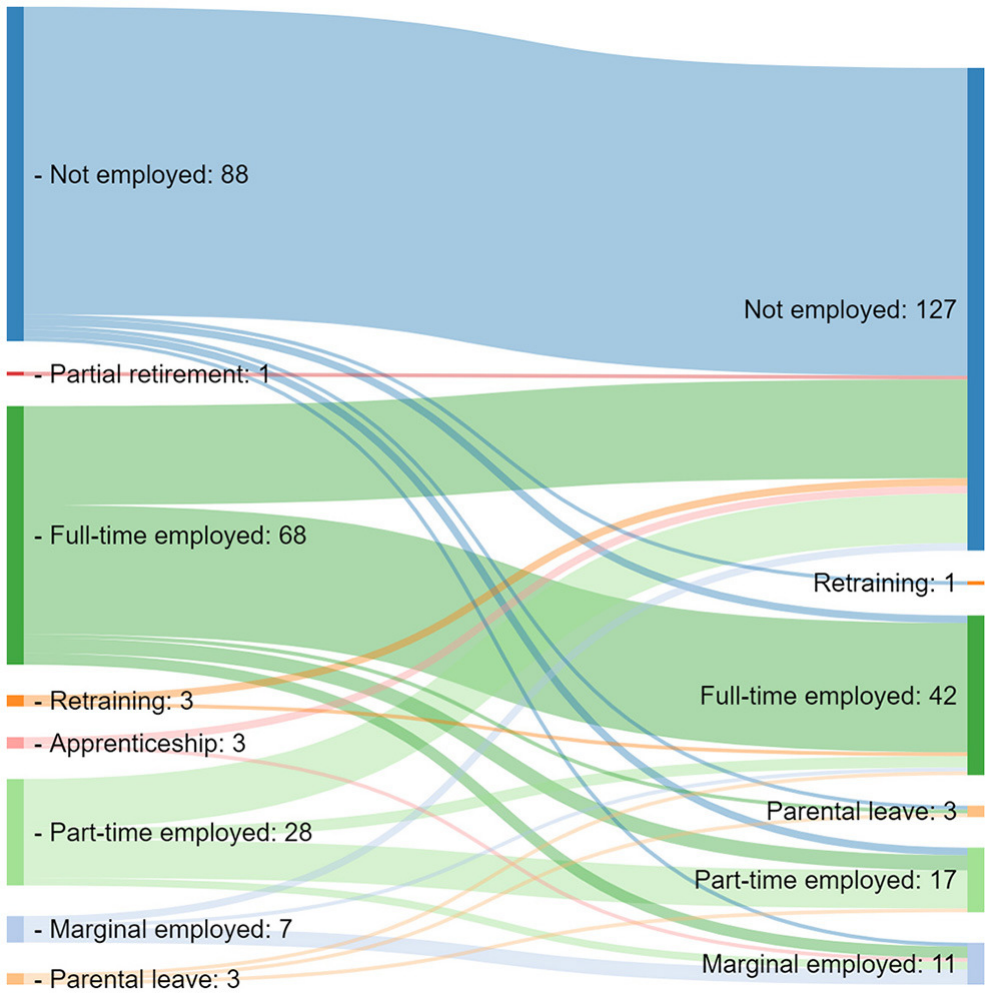
Sankey chart of changes in employment from diagnosis to study visit.

Most patients were classified as moderately educated (63%), followed by higher education of 18% and a lower educational status in 17% of the patients (Table 1). This is in line with data from the population census from Germany 2018. Here 57% were moderately educated, followed by 25% with a lower and 18% with a higher educational status, respectively. At the time of diagnosis, 44% of the patients were unemployed, 34% were employed fulltime, and 14% were employed part-time. At study visit, unemployment was determined in 63% of the patients (+19%), fulltime employment in 21% (−13%), and 8% were employed part-time (-6%) (*p* < 0.001, see Table 2 and Figure 2). Retirement was due to age (*n* = 50, 65%) or disability (*n* = 27, 35%). Compared to the German population with 6.2% of adult citizens retired due to disability, PAH patients had a significantly higher rate of retirement due to disability (*p* < 0.001). The WPAI score of employed patients at diagnosis was 6 (3–8) points, and it was 2 (0–7) points at study visit (*p* = 0.001). The WPAI score at diagnosis was available for 96 employed patients, and at study visit, *n* = 57 patients who were employed stated a WPAI score (−39 patients).

**Table 2:** Change of employment and work productivity from diagnosis to study visit.

	**Diagnosis**	**Study visit**
Item	*n* = 201, 11 missing values (5%)	
Unemployed	88 (44%)	(3%)
° Retired	(59%)	77 (61%)
•Old age pension	(33%)	50 (39%)
•Disability pension	(26%)	27 (21%)
° Disability	12 (14%)	27 (21%)
° Homekeeper	10 (11%)	9 (7%)
° Unoccupied	4 (5%)	11 (9%)
° Others	4 (5%)	1 (1%)
° College/university students	4 (5%)	1 (1%)
° Students	2 (2%)	1 (1%)
Full-time employed	68 (34%)	21%)
Part-time employed	28 (14%)	(8%)
Marginal employed	7 (3%)	(5%)
Retraining	3 (1%)	(1%)
Apprenticeship	3 (1%)	(0%)
Parental leave	3 (1%)	(1%)
Partial retirement	1 (1%)	0 (0%)
**WPAI, median (IQR)**	6 (3–8)	2 (0–7)—*p* = 0.001

*IQR, interquartile range; WPAI, work productivity and impairment*.

The EQ-5D-3L score was 7 (6–9), EQ-VAS was 5.5 (3.5–7.5), and emPHasis-10 was 18 (13–27) points at study visit. The EQ-5D-3L as well as emPHasis-10 showed more problems in physical sub-scores. In the dimension mobility of EQ-5D-3L, 55% had some or extreme problems; in the dimension pain/discomfort, 80% had moderate or extreme problems, while 39% had some or extreme problems in the dimension anxiety/depression. The sub-scores of emPHasis-10 showed the most problems in sub-score 6 (breathlessness in walking up a flight of stairs), with a median of 3 (1–4) points, and in sub-score 5 (having lots of energy), with a median of 3 (2–4) points. Least problems in emPHasis-10 were found in sub-score 2 (interrupting conversations due to breathlessness), with a median of 1 (0–2) points, and sub-score 7 (confidence in public places), with a median of 1 (0–3) points. In EQ-5D-3L, least problems were found in the dimension self-care, with 70% of the patients stating no problems.

Comparing the QoL for employed vs. unemployed patients at study visit, the EQ-5D-3L score was 9 (8–10) vs. 6 (5–8) points (*p* < 0.001), EQ-VAS was 7.5 (5–8.5) vs. 4.5 (3–6) points (*p* < 0.001), and emphasis-10 was 15 (8–24) vs. 21 (14–29) points (*p* = 0.001), respectively. In the EQ-5D-3L dimension mobility, 24% of employed patients had no problems vs. 71% of unemployed patients (*p* < 0.001). In the dimension anxiety/depression, 23% of employed patients stated problems, while 49% of patients who were not employed had problems in this dimension (*p* < 0.001).

### Impact on Employment Status

This analysis included 162 patients eligible of being employed (age at study visit, <67 years). Univariate models were calculated using the following predictors of being employed: education (low, moderate, and high), female sex, smoking (never a smoker, former smoker, and active smoker), flight of stairs, WHO FC, PFT (FEV_1_ and DLCO), Audit-C, ESC/ERS risk score, EQ-VAS, EQ-5D-3L, emPHasis-10, and degree of disability on the odds of being employed. The significant covariates education (low, moderate, and high), formerly smoking, flight of stairs, WHO FC, 6MWD per 50-m increase (22), PFT (FEV_1_ and D_LCO_), Audit-C, ESC/ERS-Risk, NT-proBNP as ≥300 ng/L (23), EQ-VAS, EQ-5D-3L, and emPHasis-10 were included in the multivariate regression model. Moderate (vs. low) education had an OR of 3.6 (1.2–11.3), *p* = 0.025 being employed, and high education had an OR of 5.6 (1.3–23.4), *p* = 0.019 of being employed. In addition, 6MWD was associated with being employed, with an OR of 1.2 (1.0–1.3), *p* = 0.042 per 50-m increase (see Table 3).

**Table 3:** Influencing factors on employment in pulmonary arterial hypertension patients.

	**Univariate**		**Multivariate**	
**Parameter**	OR (95% CI)	*p*	OR (95% CI)	*p*
Education				
Low	1 (reference)	<0.001	1 (reference)	0.042
Moderate	3.7 (1.6–8.9)	0.003	3.6 (1.2–11.3)	0.025
High	10.4 (3.4–32)	<0.001	5.6 (1.3–23.4)	0.019
**Female sex**	0.8 (0.4–1.5)	0.481		
**Age at diagnosis per 5 years increase**	0.8 (0.7–0.9)	<0.001		
Smoking				
Never a smoker	1 (reference)			
Former smoker	2.6 (1.4–4.9)	0.004		
Active smoker	1.4 (0.6–3.3)	0.385		
**Stair climbing (flights of stairs)**	2.0 (1.4–2.8)	<0.001		
**WHO FC**	0.4 (0.3–0.6)	<0.001		
**6MWD**_**50mincrease**_	1.5 (1.2–1.7)	<0.001	1.2 (1.0–1.3)	0.042
**Number of PH medications**	1.1 (0.7–1.6)	0.816		
**FEV**_**1**_ **%**	1.0 (1.0–1.0)	0.016		
**D**_**LCO**_ **%**	1.0 (1.0–1.1)	<0.001		
**ESC/ERS risk score**	0.4 (0.2–0.6)	<0.001		
**NT-proBNP**, **≥300 ng/L**	0.74 (0.37–1.48)	0.394		
**EQ-VAS**	1.3 (1.1–1.4)	0.001		
EQ-5D-3L	2.3 (1.7–3.0)	<0.001		
Mobility dimension	8.0 (3.9–16.7)	<0.001		
Self-care dimension	6.1 (2.4–15.4)	<0.001		
Usual activities dimension	8.1 (3.9–16.7)	<0.001		
Pain/discomfort dimension	6.9 (2.9–16.3)	<0.001		
Anxiety/depression dimension	2.4 (1.3–4.5)	0.007		
**emPHasis-10**	1.2 (1.0–1.4)	0.022		
Degree of disablement	1.2 (0.6–2.2)	0.579		

*OR, odds ratio; CI, confidence interval; WHO-FC, World Health Organization functional class; FEV1, forced expiratory volume in 1 s; DLCO, diffusing capacity of the lung for carbon monoxide; AUDIT-C, Alcohol Use Disorder Identification Test; ESC, European Society of Cardiology; ERS, European Respiratory Society; NT-proBNP, N-terminal fragment of probrain natriuretic peptide; 6MWD, 6-min walk distance; VAS, Visual Analogue Scale*.

## Discussion

This present study examined the impact of PAH on employment status and quality of life. We demonstrated (i) the influence of educational status and 6MWD on employment status, (ii) good work productivity in long-term-employed patients, and (iii) a considerable loss of employment with associated limited social participation and subsequent unfavorable QoL in patients with PAH patients.

In the present study, we demonstrated the impact of educational status and 6MWD on employment status in patients with PAH. Long-term-employed patients had good work productivity, whereas impaired work productivity and employment rates were associated with limited social participation and, subsequently, worse QoL.

Overall, the employment rates in our study were in line with a study from Matura et al. (10), whereas in subgroup analyses they revealed higher rates of retirement due to disability compared to our study. These differences may be partly related to disparities in the US and German healthcare systems. In Germany, the legal retirement age is stepwise increasing from 65 to 67 years. In addition, after a certain age-adjusted time on disability leave, people enter retirement plans. In this study, the rate of retirement due to disability in patients with PAH was substantially higher than in the general German population, which is not an unexpected finding in a debilitating disease.

### Factors Influencing Employment

To our knowledge, the impact of PAH on employment and its influencing factors have not been investigated in Germany to date. Our study found that, compared to patients with lower education, patients with non-academic education (moderate education category*)* had a 3.6-fold higher chance of being employed, whereas patients with academic education (high education category) had a 5.6-fold chance of being employed. This might be explained by the association of physical labor and lower education. Accordingly, highly educated individuals in white-collar jobs are less likely to experience manual labor (7), and this might contribute to a higher likelihood of employment for PAH patients with a higher educational status despite the burden of the disease. However, we cannot rule out that education might have an impact on employment itself, and further studies are needed to answer this question.

The severity of PAH related to physical impairment is, in part, described by 6MWD (24, 25). In our study, we found that a higher 6MWD was associated with employment status. Inversely, we can conclude that physical impairment is a major risk factor for unemployment. No other disease severity parameter was included in the multivariate model, and we presume that this was due to the multicollinearity of disease severity parameters (WHO FC, NT-proBNP, and ESC/ERS risk status).

Matura et al. demonstrated that education and physical status influence the QoL in PAH (10). In our study, we assessed associations of employment using education and QoL as independent factors. In the univariate analysis, a higher QoL was associated with employment, which is in line with the findings of Matura et al. Furthermore, Helgeson et al. found in a study regarding the psychosocial and financial burden of therapy in the USA that 53% of initially working patients were no longer employed (26). Compared to the 61% loss in full- or part-time employment in our study, the differences might be explained by the different social security systems in the US and Germany.

Cascino et al. revealed associations between socioeconomic status and the perceived need for exercise rehabilitation in patients with PAH (27). Patients with a lower socioeconomic status had a significant lack of perceived need for rehabilitation, were less informed about the existence of exercise therapy, and preferred to take care of their health alone. We acknowledge the need to explore interventions to promote referral especially among low-educational-status patients to improve their exercise capacity as we see such a clear impact on employment.

In our study, there were strong associations between QoL, education, employment status, and physical condition. Furthermore, we showed that the employed patients had a significantly better QoL in EQ-5D-3L, EQ-VAS, and emphasis-10 in contrast to unemployed patients. Olsson et al. described a high prevalence of mental disorders in 38% of PAH patients, with a negative impact on QoL (28). We showed similar results with 39% of PAH patients having an impaired EQ-5D-3L dimension anxiety/depression. Undoubtedly, PAH has a direct impact on physical impairment and, *via* this pathway, on mental disorders like anxiety or depression, resulting in reduced QoL, employment rate, and work productivity in this patient group.

PAH remains a partly “invisible” disease as Kingman et al. described the perspective of patients to feelings of insecurity and isolation (29). This was mainly driven by the lack of understanding of the disease and symptoms among family, friends, and the general public. Kingsman et al. found that different coping strategies played a major role. Patients who sought solutions tried to maintain a social life. With more independence, they were, in many cases, able to work at least part-time. Furthermore, these authors proposed a greater psychological impact of PH on the QoL of patients, emphasizing the importance of healthcare providers and physicians to educate the patients on disease progression, symptoms, and effects on QoL. In our work, we did not assess the coping strategy of patients, but the impact of the coping strategies should be addressed in further research. In this and previous studies (Olsson et al.), we did see associations between QoL and mental health, leading us to believe that encouraging participation in support groups, PH associations, and counseling should be an aspect addressed in the healthcare provider and patient relationship.

### Limitations

Our study had several limitations, including the cross-sectional design and the participation of two study sites. Prospective studies with a structured follow-up are needed to assess the impact of the disease over time. Paper-based questionnaires led to missing data in some cases. Data on physical/manual labor or job descriptions, to assess the assumed surrogate effect of educational status, were missing. Although we assessed mental disorders as part of the EQ-5D-3L, future research should consider utilizing dedicated mental health questionnaires, e.g., the Hospital Anxiety and Depression Scale, as suggested by Olsson et al. (28). The strengths of our study were the high number of surveyed patients and the important but understudied issue of social participation of PAH patients.

## Conclusion

Employment and work productivity in patients with PAH were impaired, and there was a considerable loss of employment during the course of the disease. Education and 6MWD were the relevant factors associated with employment in patients with PAH. The employed patients had a significantly better QoL than the unemployed patients. Patients in physical or manual labor were more likely to be unemployed. Loss of employment had a major impact on the life and mental health of patients. Re-education strategies are needed for patients who are eligible to work and should be an aspect of empowerment from their treating physicians. Employment can provide patients with more independence and thus a more favorable QoL and social participation.

## Data Availability Statement

The raw data supporting the conclusions of this article will be made available by the authors, without undue reservation.

## Ethics Statement

The studies involving human participants were reviewed and approved by Ethikkommission der Medizinischen Hochschule Hannover. The patients/participants provided their written informed consent to participate in this study.

## Author Contributions

JF was responsible for study design, implementation of the study, data collection, statistical analysis, data interpretation, and drafting the manuscript. DHP was responsible for implementation of the study, data collection, statistical analysis, data interpretation, and drafting the manuscript. TvL was responsible for study design, data interpretation, and critically revising the manuscript. HGa, MR, and HGh were responsible for implementation of the study, data collection, and revising the manuscript. JK was responsible for data interpretation and revising the manuscript. MH was responsible for study design, implementation of the study, data interpretation, and critically revising the manuscript. KO was responsible for study design, implementation of the study, data interpretation, and critically revising the manuscript. All authors contributed to the article and approved the submitted version.

## Conflict of Interest

HGa has received personal fees from Actelion, AstraZeneca, Bayer, BMS, GSK, Janssen-Cilag, Lilly, MSD, Novartis, OMT, Pfizer, and United Therapeutics, outside the submitted work. HGh has received fees from Actelion, Bayer, Gilead, GSK, MSD, Pfizer, and United Therapeutics, outside the present work. MH has received fees for lectures and/or consultations from Acceleron, Actelion, Bayer, GSK, Janssen, MSD, and Pfizer, all outside the present work. KO has received fees for lectures and/or consultations from Acceleron, Actelion, Bayer, Janssen, MSD, United Therapeutics, GSK, and Pfizer, all outside the present work. The remaining authors declare that the research was conducted in the absence of any commercial or financial relationships that could be construed as a potential conflict of interest.

## Publisher's Note

All claims expressed in this article are solely those of the authors and do not necessarily represent those of their affiliated organizations, or those of the publisher, the editors and the reviewers. Any product that may be evaluated in this article, or claim that may be made by its manufacturer, is not guaranteed or endorsed by the publisher.

## Abbreviations

6MWD: 6-min walk distance
APAH: associated PAH
AUDIT-C: Alcohol Use Disorder Identification Test
BMI: body mass index
BNP: brain natriuretic peptide
DLCO: diffusing capacity of the lung for carbon monoxide
ERS: European Respiratory Society
ESC: European Society of Cardiology
FC: World Health Organization functional class
FEV1: forced expiratory volume in 1 s
HADS: Hospital Anxiety and Depression Scale
HPAH: hereditary PAH
HRQoL: quality of life
IPAH: idiopathic PAH
NT-proBNP: N-terminal fragment of probrain natriuretic peptide
OR: odds ratio
PAH: pulmonary arterial hypertension
PCH: pulmonary capillary hemangiomatosis
PFT: pulmonary function testing
PH: pulmonary hypertension
PVOD: pulmonary veno-occlusive disease
QoL: quality of life
SD: standard deviation
VAS: Visual Analog Scale
WPAI: work productivity and impairment.
