# Supplementary material for: Impact of Pulmonary Arterial Hypertension on Employment, Work Productivity, and Quality of Life - Results of a Cross-Sectional Multi-Center Study
Source: Front Psychiatry. 2022 Feb 8;12:781532. doi: 10.3389/fpsyt.2021.781532 (PMC8861193; doi:10.3389/fpsyt.2021.781532)
Supplement: Supplementary file 1 [file Table_1.pdf]

Table S1 – Instruments used for the assessment of QoL

	EQ-5D-3L	EQ-VAS	EMPHASIS-10
**Questions**	5	1	10
**Scale per question**	0-2 points in 1.0 steps	0 – 10 in 0.5 steps	0-5 points in 1.0 steps
**Scale-type**	Likert Scale	VAS	Likert Scale
**Scoring**	Summation	-	Summation
**Total score level**	0-10 points	0-10 points	0-50 points
**Interpretation**	Higher is better QoL	Higher is better QoL	Lower is better QoL
**Domains**	5 (mobility, self-care, usual activities, pain/discomfort and anxiety/depression)	1 (general condition)	2 (physical restraints, psychosocial aspects)

ABBREVIATIONS: QOL, QUALITY OF LIFE; VAS, VISUAL ANALOGUE SCALE; EQ-5D-3L, EUROQOL 5 DIMENSIONS QUESTIONNAIRE;
